# Supplementary material for: Antigen-specific CD8+ T cell feedback activates NLRP3 inflammasome in antigen-presenting cells through perforin
Source: Nat Commun. 2017 May 24;8:15402. doi: 10.1038/ncomms15402 (PMC5458103; doi:10.1038/ncomms15402)
Supplement: Supplementary Information — Supplementary Figures and Supplementary Table 1 [file ncomms15402-s1.pdf]

Supplementary Figure 1

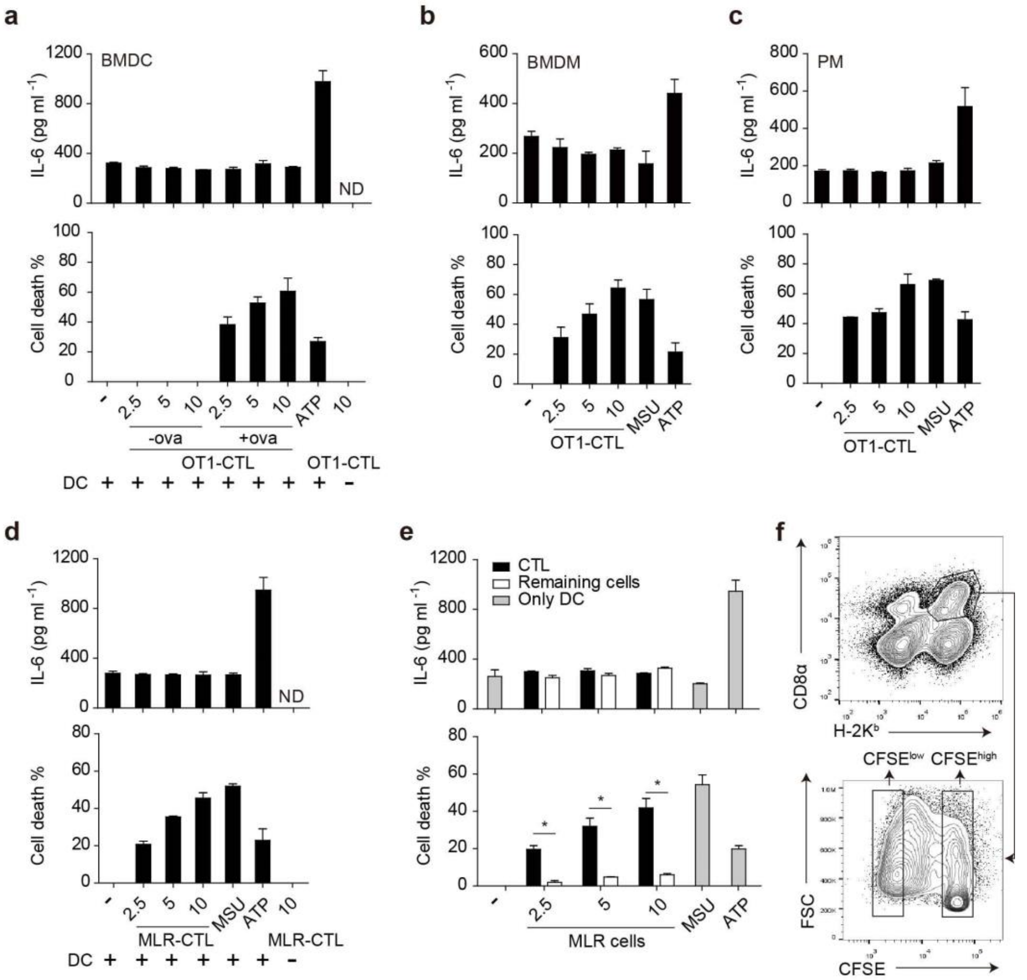

Supplementary Figure 1. Antigen-specific CTLs activate ASC inflammasome in APCs. (**a**-**c**) The level of IL-6 determined by ELISA and cell death determined by LDH release from LPS-primed and ova-pulsed BMDCs (**a**), BMDMs (**b**) or peritoneal macrophage (PM) (**c**) co-cultured with OT1-CTLs at the indicated ratios for 4 h. (**d**) The level of IL-6 and cell death from LPS-primed BMDCs co-cultured with MLR-CTLs (C57 anti-BALB/c) at the indicated ratios for 4 h. (**e**) The level of IL-6 and cell death from LPS-primed BMDCs co-cultured with MLR CTLs or all the other remaining cells (C57 anti-BALB/c) at the indicated ratios for 4 h. (**f**) Sorting strategy by CFSE dilution with the gating for H-2K^{b+} CTLs from MLR at day6. Data are representative of two (a,c,f) or three (b,d,e) independent experiments. Error bars, SEM. *$p < 0.05$ by two-tailed Student's t test.

Supplementary Figure 2

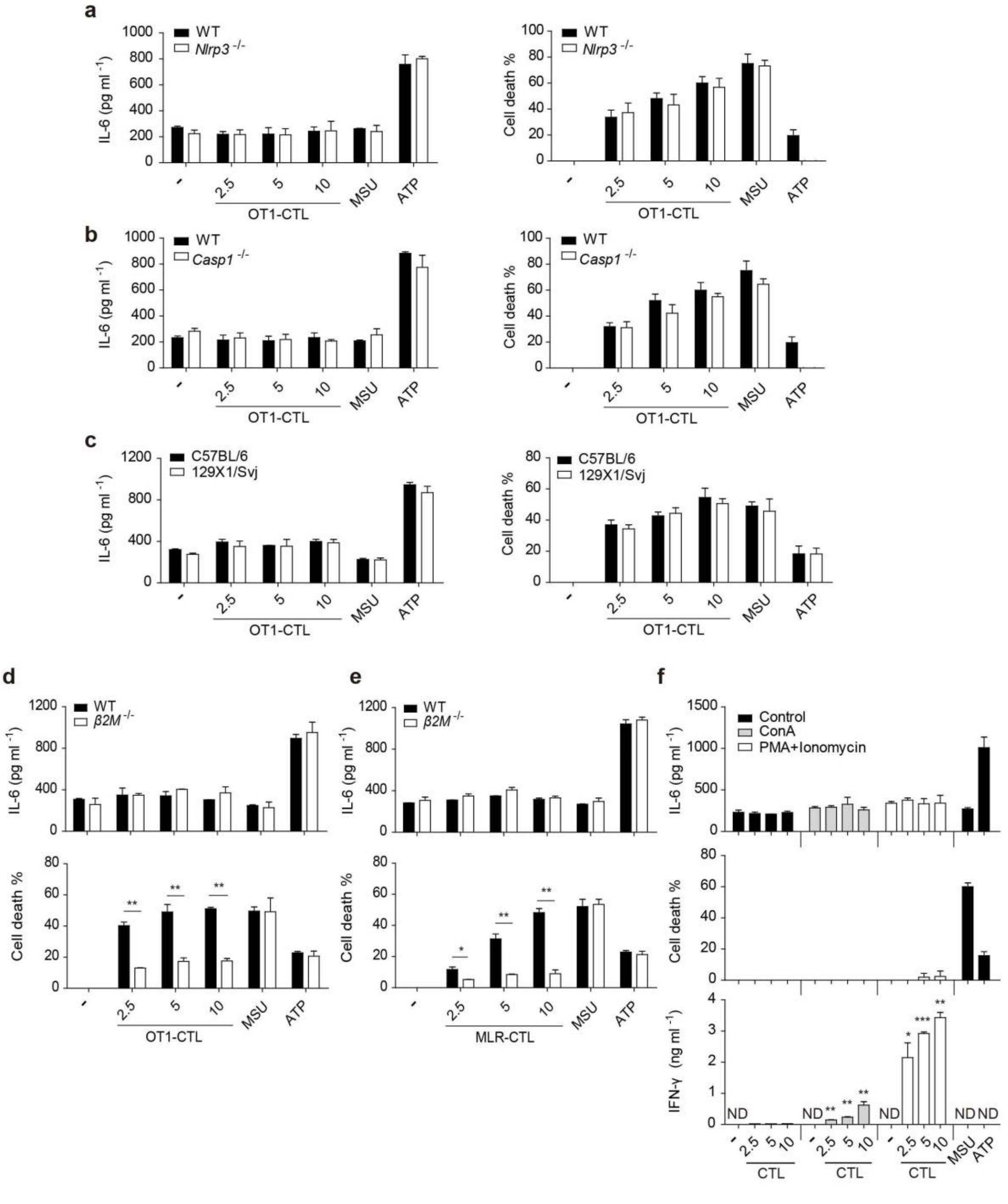

Supplementary Figure 2. NLRP3 inflammasome is required for antigen-specific CTL induced IL-1β secretion in innate APCs through MHC I. (**a**) IL-6 release and cell death from LPS-primed and ova-pulsed wild-type or *Nlrp3*$^{-/-}$ BMDCs co-cultured with OT1-CTLs. (**b**) IL-6 release and cell death from LPS-primed and ova-pulsed wild-type or *Casp1*$^{-/-}$ BMDCs co-cultured with OT1-CTLs. (**c**) The level of IL-6 and cell death from LPS-primed and ova-pulsed C57BL/6 or 129X1/SvJ BMDCs co-cultured with OT1-CTLs. (**d**, **e**) The level of IL-6 and cell death from LPS-primed and ova-pulsed wild-type or *β2M*$^{-/-}$ BMDCs co-cultured with OT1-CTLs (**d**) or MLR-CTLs (**e**). (**f**) The levels of IL-6 and IFN-γ as well as cell death from LPS-primed

BMDCs co-cultured with wild-type CTLs pre-stimulated with ConA or PMA plus ionomycin at the indicated ratios for 4 h. Data are representative of two (a-f) independent experiments. Error bars, SEM. *$p < 0.05$, **$p < 0.01$, ***$p < 0.001$ by two-tailed Student's t test.

Supplementary Figure 3

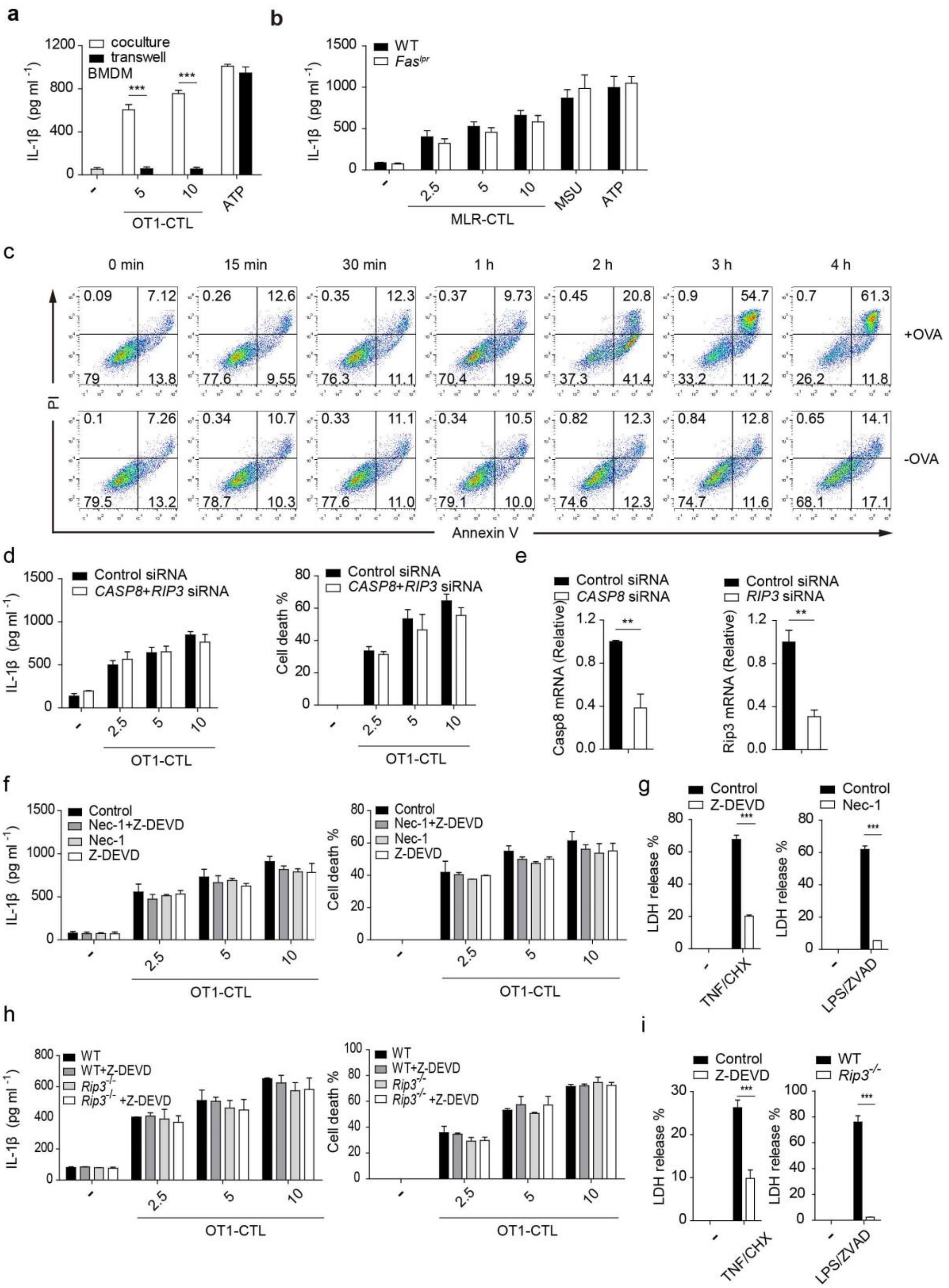

Supplementary Figure 3. Cell apoptosis and necroptosis are not required for antigen-specific CTL induced IL-1β secretion in APCs. (**a**) IL-1β release from LPS-primed and ova-pulsed BMDMs, cocultured with OT1-CTLs or with transwell-seperated OT1 CTLs for 4 hours. LPS+ATP (ATP) were added as a positive control. (**b**) IL-1β release from LPS-primed wild-type or *Fas*^{lpr} BMDCs co-cultured with MLR-CTLs for 4 h. (**c**) Flow analyses of the cell populations by annexin V/PI staining in the co-culture of LPS-primed and ova pulsed BMDCs with OT1-CTLs for the indicated time. (**d**) IL-1β release and cell death from control siRNA or CASP8 siRNA plus RIP3 siRNA treated BMDMs that were LPS-primed and ova pulsed and then co-incubated with OT1-CTLs. (**e**) Knock-down levels of CASP8 and RIP3 as in **d**. (**f**) IL-1β release and cell death from LPS-primed and ova pulsed BMDCs that were treated with apoptosis inhibitor (Z-DEVD 20μM) and necrosis inhibitor (Nec-1 20μM) and then co-incubated with OT1-CTLs. (**g**) Positive controls of the effects of apoptosis inhibitor (Z-DEVD) and necrosis inhibitor (Nec-1). (**h**) IL-1β release and cell death from LPS-primed and ova pulsed wild-type and *Rip3*$^{-/-}$ BMDMs that were treated with the apoptosis inhibitor (Z-DEVD) and then co-incubated with OT1-CTLs. (**i**) Positive controls of the effects of the apoptosis inhibitor (Z-DEVD) and *Rip3* deficiency on the relevant pathways. Data are representative of two (b,c,f,h) or three (a,d,e,g,i) independent experiments. Error bars, SEM. **$p < 0.01$, ***$p < 0.001$ by two-tailed Student's t test.

Supplementary Figure 4

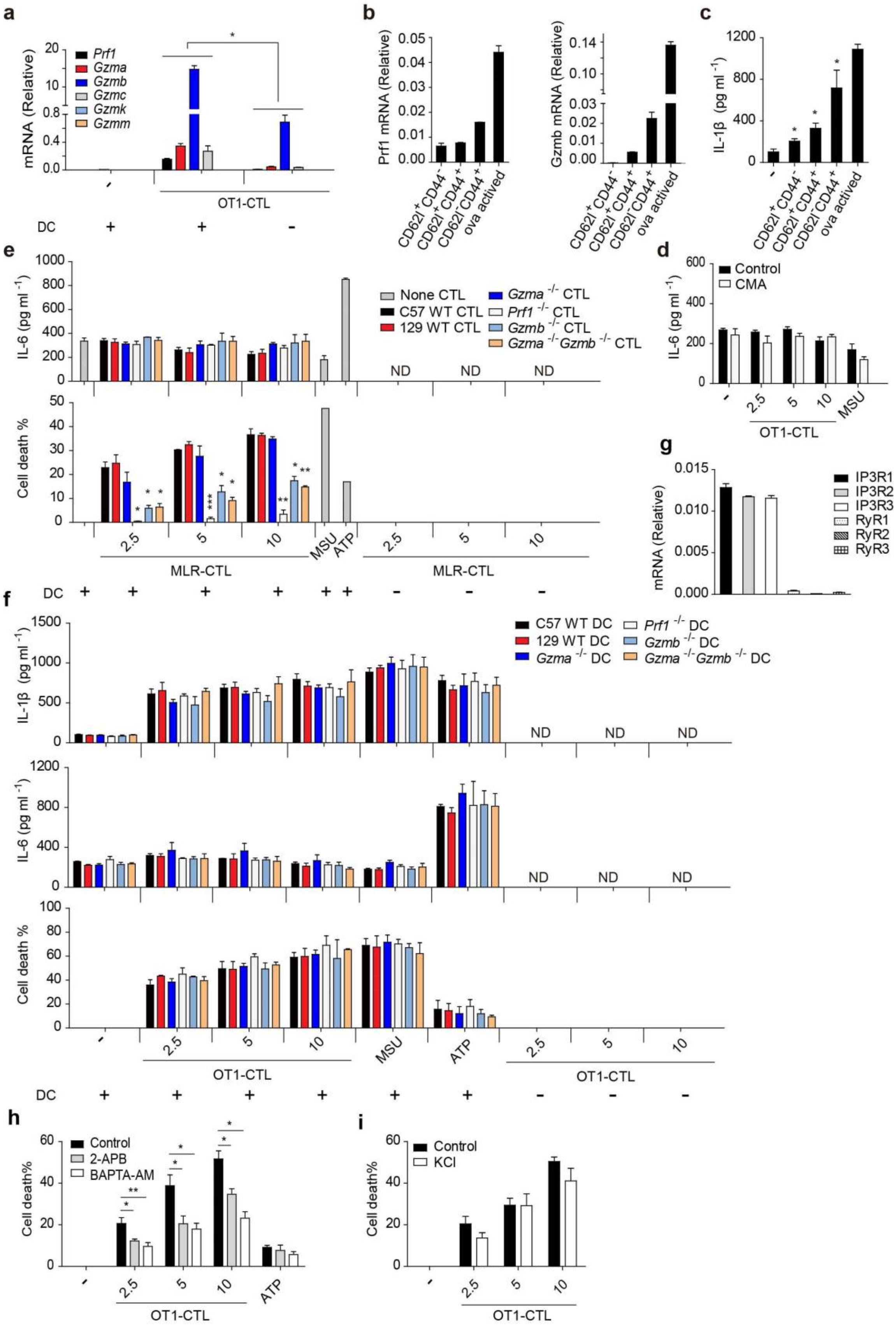

Supplementary Figure 4. Perforin in antigen-specific CTLs is critically required for IL-1β secretion in APCs. (**a**) The mRNA levels of perforin (prf1) and granzymes determined by qPCR from LPS-primed and ova-pulsed BMDCs co-cultured with or without OT1-CTLs for 2 h. (**b**) The mRNA levels of prf1 and granzyme B of different CTL populations from naïve OT1 mice or ova activated OT1 cells. (**c**) IL-1β secretion from BMDCs co-cultured with the different CTL populations from OT1 mice as in **b**. (**d**) The level of IL-6 from LPS-primed and ova-pulsed BMDCs co-cultured with OT1-CTLs for 4 h in the presence or absence of the perforin inhibitor CMA. (**e**) IL-6 release and cell death from LPS-primed BMDCs co-cultured with wild-type or the indicated gene deficient MLR-CTLs. (**f**) The levels of IL-1β, IL-6 and cell death from LPS-primed and ova-pulsed wild-type or the indicated gene deficient BMDCs co-cultured with OT1-CTLs. (**g**) The mRNA levels of Ca^{2+} channels in BMDCs. (**h**) Cell death from LPS-primed ova-pulsed BMDCs co-cultured with OT1 CTLs in the presence or absence of 2-APB (50 μM) or BAPTA-AM (25 μM). (**i**) Cell death from LPS-primed ova-pulsed BMDCs co-cultured with OT1 CTLs in the presence or absence of KCl (50mM). Data are representative of two (d-f) or three (a-c,g-i) independent experiments. Error bars, SEM. *$p < 0.05$, **$p < 0.01$, ***$p < 0.001$ by two-tailed Student's t test.

Supplementary Figure 5

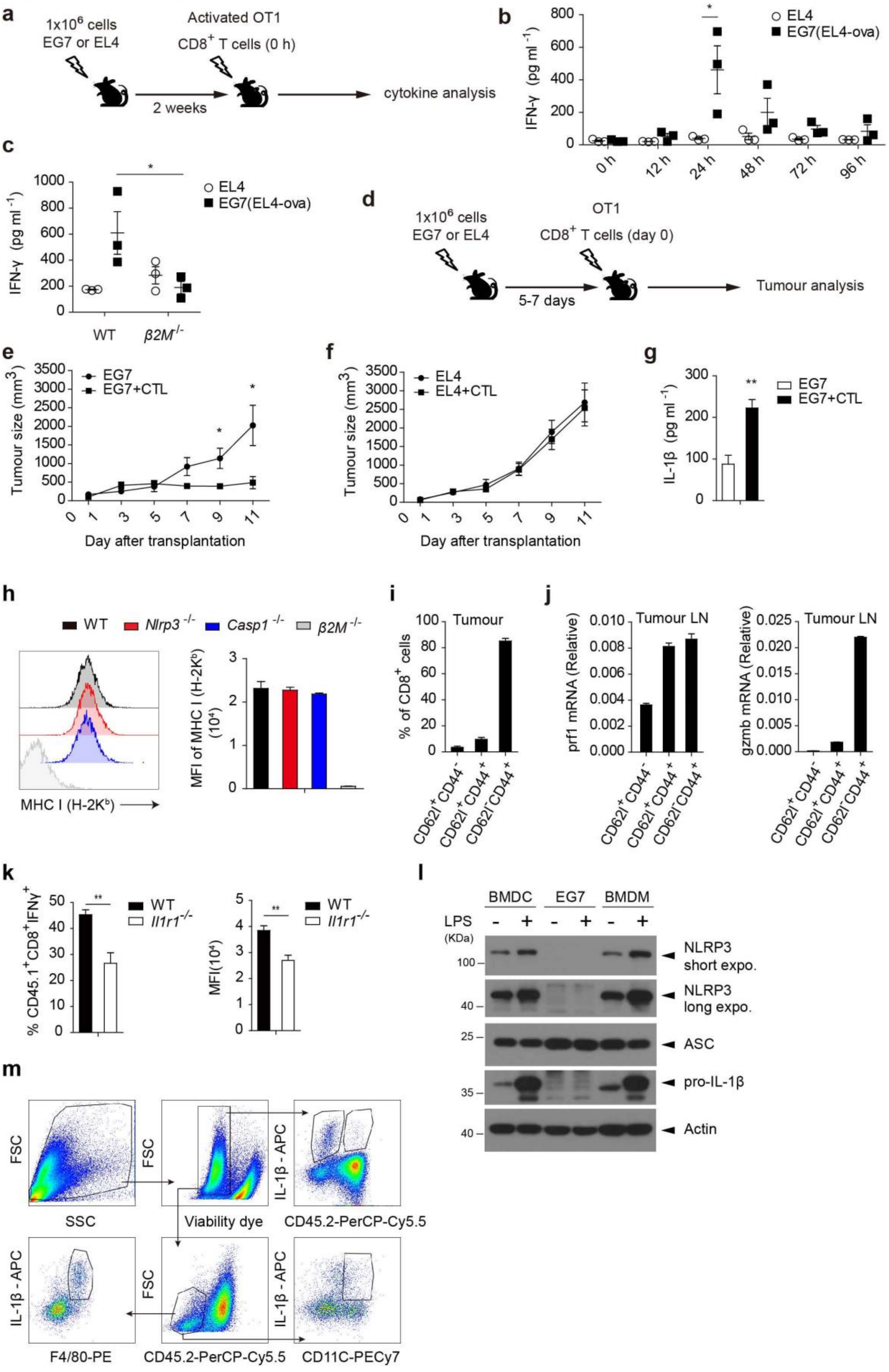

Supplementary Figure 5. Antigen specific CTL induced NLRP3 inflammasome activation in APCs contributes to the CTL mediated antitumour immunity. (**a**) The procedure used in (**b**, **c** and Fig 5**a**,**b**). Briefly, mice received s.c. injections of tumour cells (EL4 or EG7) in the right flank for about 2 weeks. After that, the mice were i.v. injected with activated OT1-CTLs and cytokines were analyzed at the indicated time. (**b**) IFN-γ secretion in tumour homogenates from wild-type mice first injected s.c. EL4 or EG7 (EL4-ova) tumour cell lines and then injected i.v. with activated OT1-CTLs. n=3 per group. (**c**) IFN-γ secretion in tumour homogenates from wild-type and *β2M*$^{-/-}$ mice first injected s.c. EL4 or EG7 (EL4-ova) cells and then injected i.v. with activated OT1-CTLs for 24 h. n=3 per group. (**d**) The procedure used in (**e**-**k** and Fig. 5**c**-**k**, 5**m**-**p**). (**e**, **f**) Tumour sizes in wild-type mice first injected s.c. with EG7 (EL4-ova) (**e**) or EL4 (**f**) tumour cell lines and then injected i.v with OT1-CTLs for the indicated time. n=5 per group. (**g**) IL-1β secretion in tumour homogenates from wild-type mice first injected s.c. with EG7 tumour cells and then injected i.v with OT1-CTLs for 5 days. n=5 per group. (**h**) The Mean Fluorescence Intensity (MFI) of MHC I (H-2K^{b}) was assessed of DCs from draining lymph nodes (LN) of wide-type, *Nlrp3*$^{-/-}$, *Casp1*$^{-/-}$or *β2m*$^{-/-}$ mice that were first injected s.c. with EG7 cells and then injected i.v with OT1-CTLs. n=3 per group. (**i**) The percentages of CTL subsets out of tumours in **g**. (**j**) The mRNA levels of perforin and granzyme B in CTL subsets of draining LN in the tumour model in **g**. (**k**) The percentage of CD45.1^{+}CD8^{+}IFN-γ^{+} T cells and MFI of IFN-γ in the CD45.1^{+}CD8^{+}IFN-γ^{+} T cells in the tumours of wild-type and *Il1r1*$^{-/-}$ mice injected i.v with CD45.1^{+} OT1 CTLs. n=5 per group. (**l**) Western blot analysis of inflammasome components in APCs (BMDC or BMDM) and EG7 tumour cells. (**m**) The FACS gating strategy presented in Fig. 5**m**-**p**. Data are representative of two (i-m) or three (b,c,e-h) independent experiments. Error bars, SEM. *p < 0.05, **p < 0.01 by two-tailed Student's t test.

Supplementary Figure 6

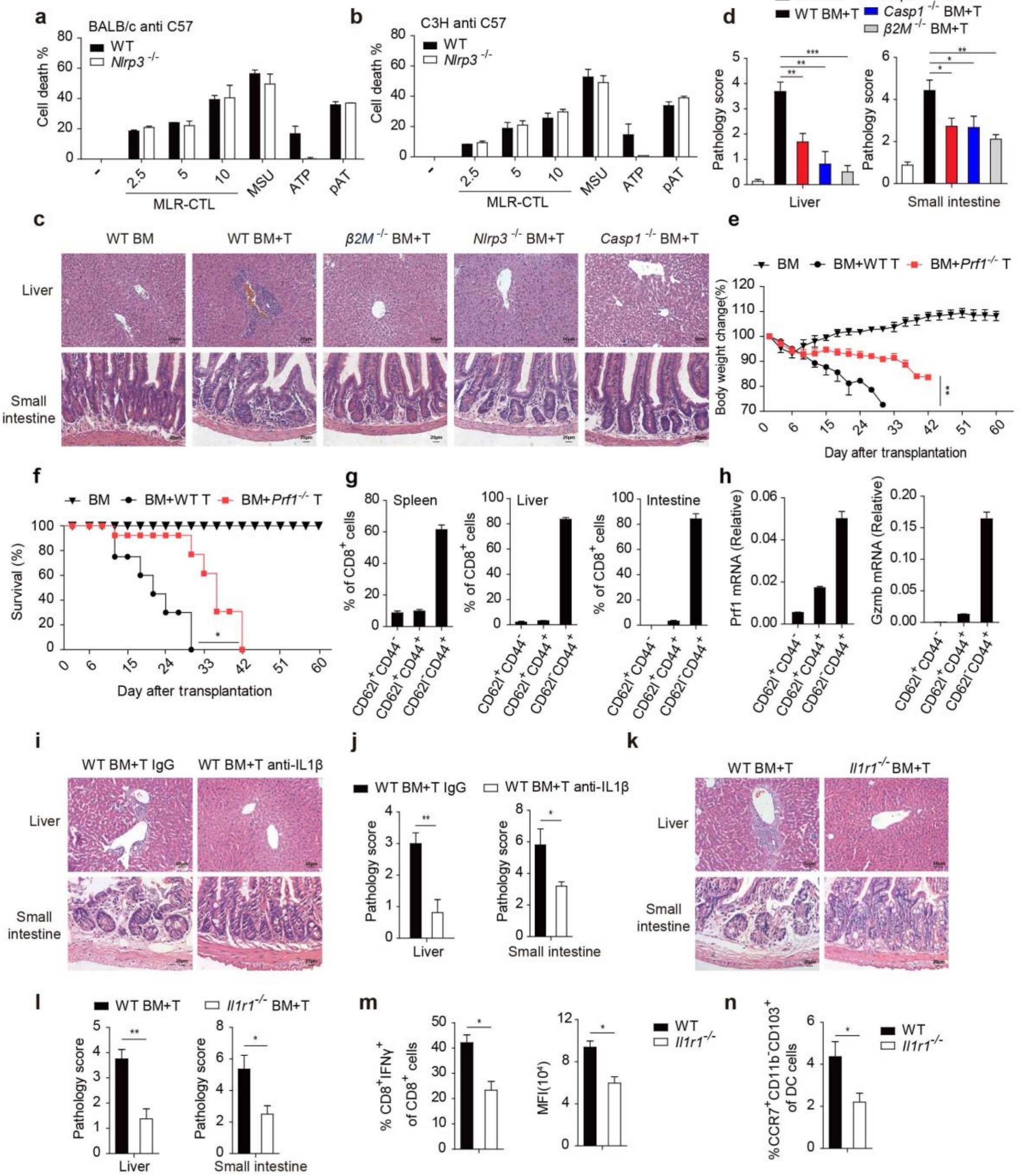

Supplementary Figure 6. Antigen specific CTL induced NLRP3 inflammasome activation in APCs contributes to the CTL mediated GVHD pathogenesis. (**a**, **b**) Cell death determined by LDH release of LPS-primed wild-type or *Nlrp3*$^{-/-}$ BMDCs co-cultured with BALB/c anti-C57 (**a**) or C3H anti-C57 (**b**) MLR-CTLs for 4 h. (**c**) H&E staining of liver and small intestine from wild-type, *Nlrp3*$^{-/-}$, *Casp1*$^{-/-}$ or *β2M*$^{-/-}$ mice underwent total body irradiation (TBI) followed by i.v. injection with C3H BM (bone marrow) alone or C3H BM plus CTLs for 35 days. Scale bars, 50 μm (liver), 20 μm (small intestine). (**d**) Pathological scores of liver and small intestine samples as in **c**. n=6 per group. (**e**,**f**) The level of body weight change (e) and survival rate (f) of mice underwent TBI followed by i.v. injection of bm1 BM alone or bm1 BM plus CTLs from wide-type or *Prf1*$^{-/-}$ mice. n = 6 per group. (**g**) The percentage of CTL subsets in the indicated tissues at the onset (day 24) of GVHD disease. n=5 per group. (**h**) The mRNA levels of perforin and granzyme B in CTL subsets as in **g**. (**i**) H&E staining of liver and small intestine from control IgG and anti-IL1β treated mice underwent TBI followed by i.v. injection with C3H BM alone or C3H BM plus CTLs for 35 days. Scale bars, 50 μm (liver), 20 μm (small intestine). (**j**) Pathological score of liver and small intestine samples as in **i**. (**k**) H&E staining of liver and small intestine samples from wild-type, *Il1r1*$^{-/-}$ mice underwent TBI followed by i.v. injection with C3H BM alone or C3H BM plus $CD8^+$ T cells for 35 days. Scale bars, 50 μm (liver), 20 μm (small intestine). (**l**) Pathological score of liver and small intestine samples as in **k**. n=6 per group. (**m**) The percentage of $CD8^+IFN\text{-}\gamma^+$ T cells in the MLN of wild-type and *Il1r1*$^{-/-}$ mice at the onset of GVHD disease, and Mean fluorescence intensity (MFI) of IFN-γ in $CD8^+IFN\text{-}\gamma^+$ T cells. n=4 per group. (**n**) The percentage of $CCR7^+CD11b^-CD103^+$ DC cells in the intestine of wild-type and *Il1r1*$^{-/-}$ mice at day 5 post-BMT. n=8 per group. Data are representative of two (a-d,i-n) or three (e-h) independent experiments. Error bars, SEM. two-tailed Student's t test (d,j,l-n), two-way ANOVA (e), log-rank test (f). *p < 0.05, **p < 0.01, ***p < 0.001.

Supplementary Figure 7

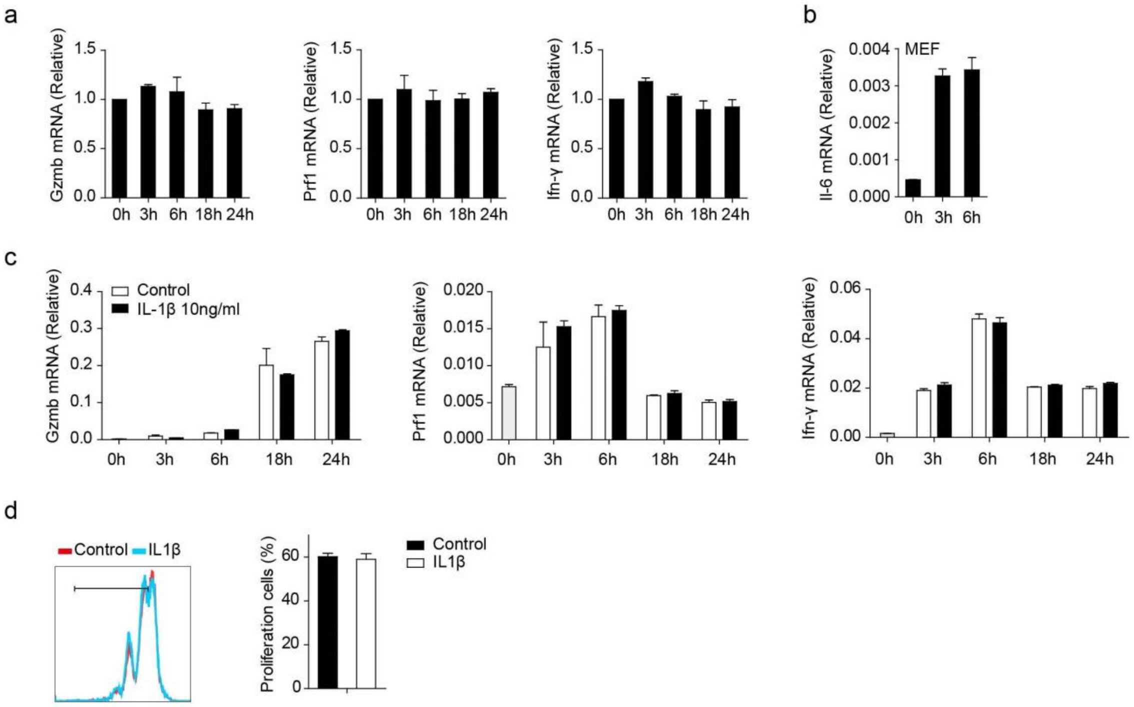

Supplementary Figure 7. IL-1β cannot directly act on CTLs for their activation. (**a**) The mRNA level of indicated genes after IL-1β (10ng/ml) stimulated on purified CTLs. (**b**) The IL-6 mRNA level in MEF after stimulated with IL-1β (positive control). (**c**) The mRNA level of indicated genes after anti-CD3 plus anti-CD28 stimulated on purified CTLs in the presence or absence of IL-1β. (**d**) CFSE intensity in CTL cells after stimulation with anti-CD3 plus anti-CD28 in the presence or absence of IL-1β, representing CTL cell proliferation. Data are representative of two (a-d) independent experiments. Error bars, SEM.

Supplementary Figure 8

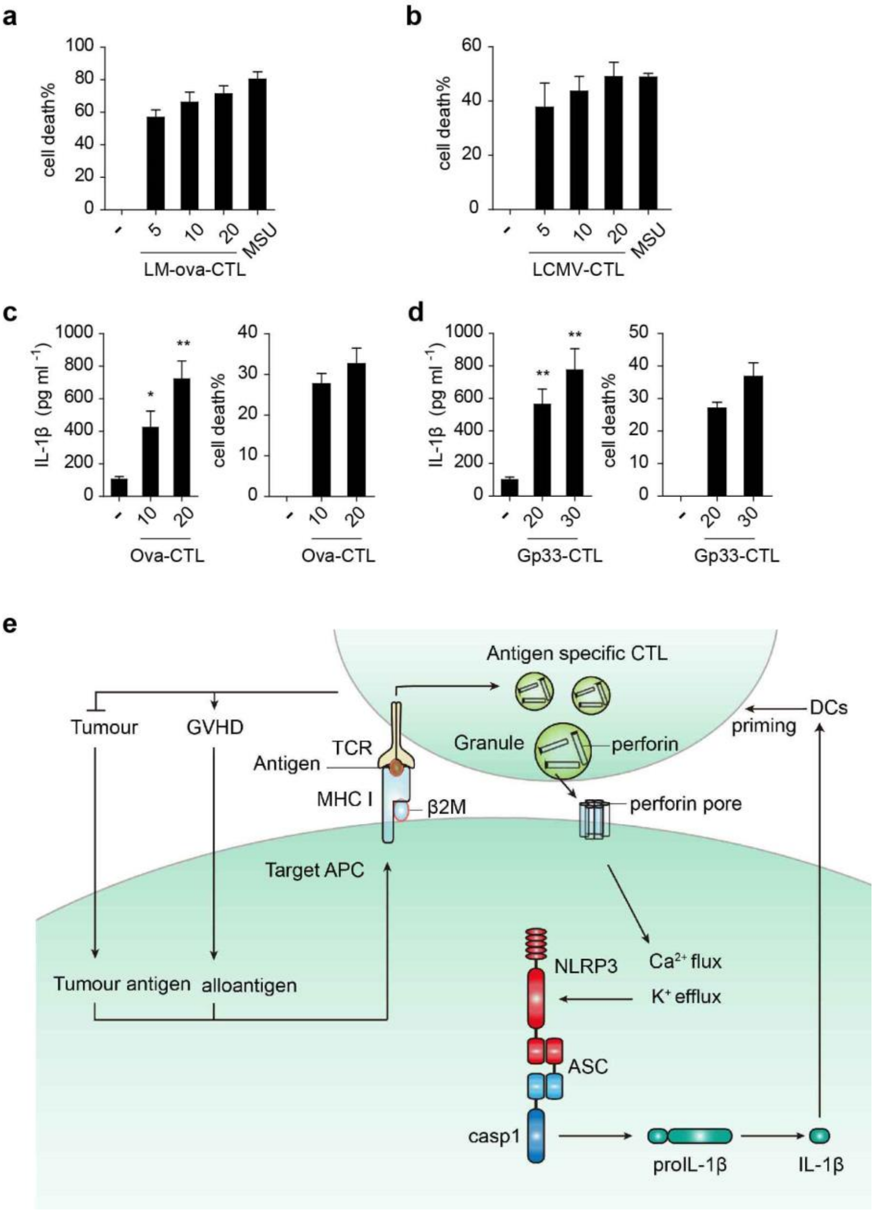

Supplementary Figure 8. Antigen specific CTL induced NLRP3 inflammasome activation in APCs contributes to the CTL mediated antitumour immunity and GVHD but not anti-virus and anti-intracellular bacterial infection. (**a**) Cell death in ova-plused BMDCs after co-cultured with CTLs from *Listeria Monocytogenes*-ova immunized mice (LM-ova-CTL) as described in methods at the indicated ratios for 4h. (**b**) Cell death in gp33-plused BMDCs after co-cultured with CTLs from LCMV immunized mice (LCMV-CTL) as described in methods at the indicated ratio for 4h. (**c**) IL-1β secretion and cell death in ova-plused BMDCs after co-cultured with ova-CTLs that from ova immunized mice for one month. (**d**) IL-1β secretion and cell death in gp33-plused BMDCs after co-cultured with gp33-CTLs that from gp33 immunized mice for one month. (**e**) The model for antigen specific $CD8^+$ T cells (CTLs) feedback activating NLRP3 inflammasome in APCs. Tumour antigens or alloantigens are processed in APCs such as dendritic cells (DCs) and presented through MHC I to TCR

in CTLs, leading to activation of antigen specific CTLs and their mediated killing of tumour cells and GVHD target cells respectively. The activated antigen specific CTLs also trigger the release of the effector molecule perforin to APCs by direct cell-cell contact. Perforin forms membrane pores and then triggers NLRP3 inflammasome activation for IL-1β maturation through calcium flux and potassium efflux in APCs. The secreted IL-1β then activates DCs to further promote functions of the CTLs. Thus, innate immunity driven antigen specific CTLs feedback promote NLRP3 inflammasome activation for IL-1β maturation in APCs to amplify antigen specific antitumour immunity and GVHD pathogenesis. Data are representative of two (a,b) or three (c,d) independent experiments. Error bars, SEM. $*p < 0.05$, $**P < 0.01$ by two-tailed Student's t test.

Supplementary Figure 9. (continued below)

Figure 1c

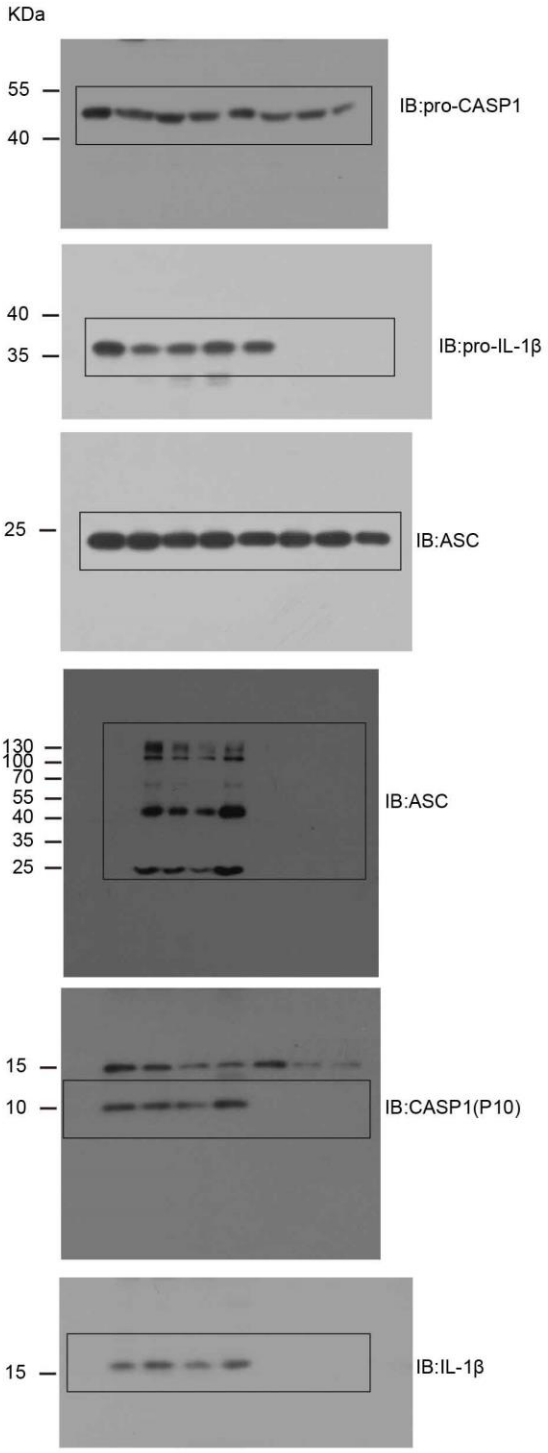

Figure 1i

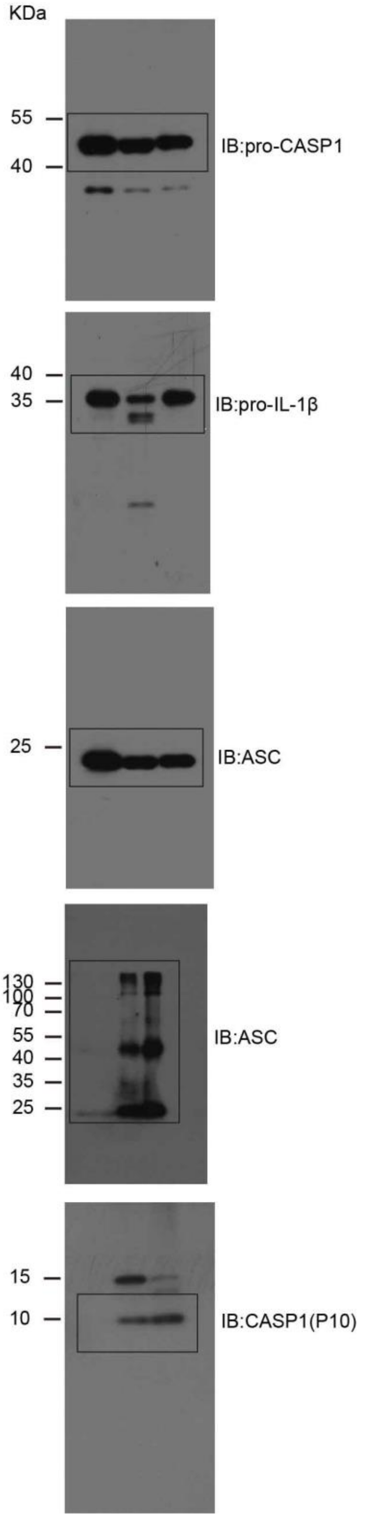

Supplementary Figure 9. (continued below)

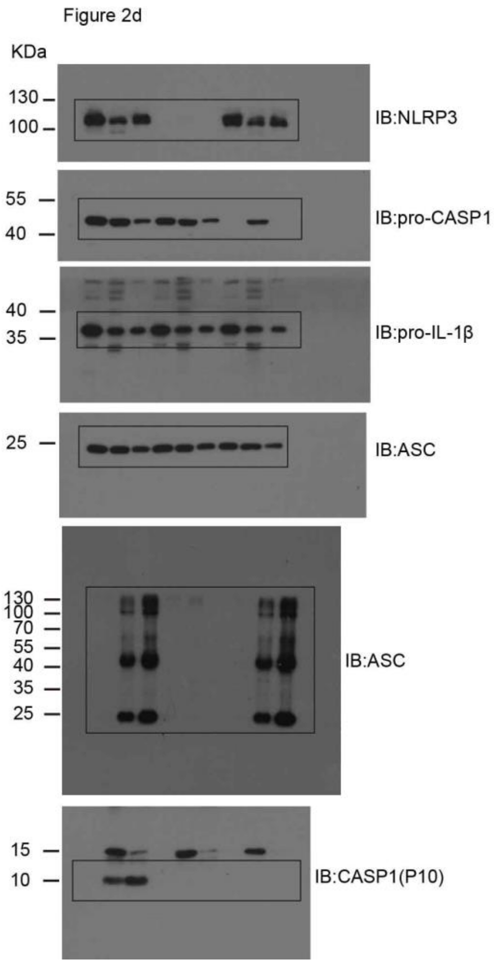

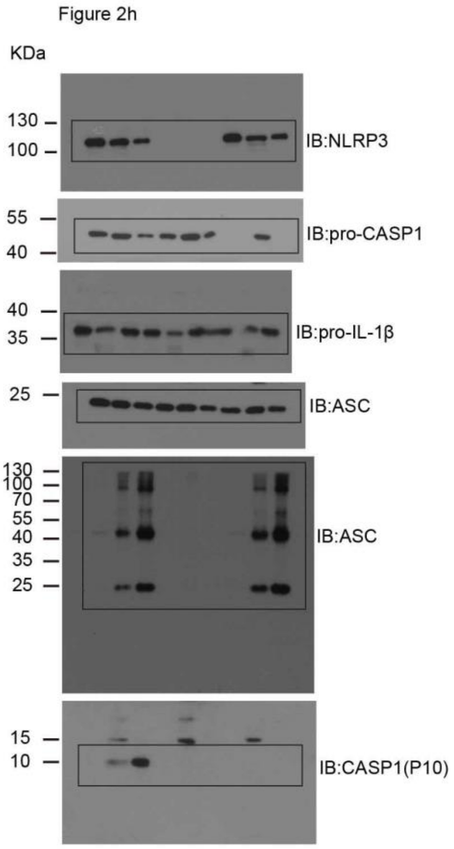

Supplementary Figure 9. (continued below)

Figure 3b

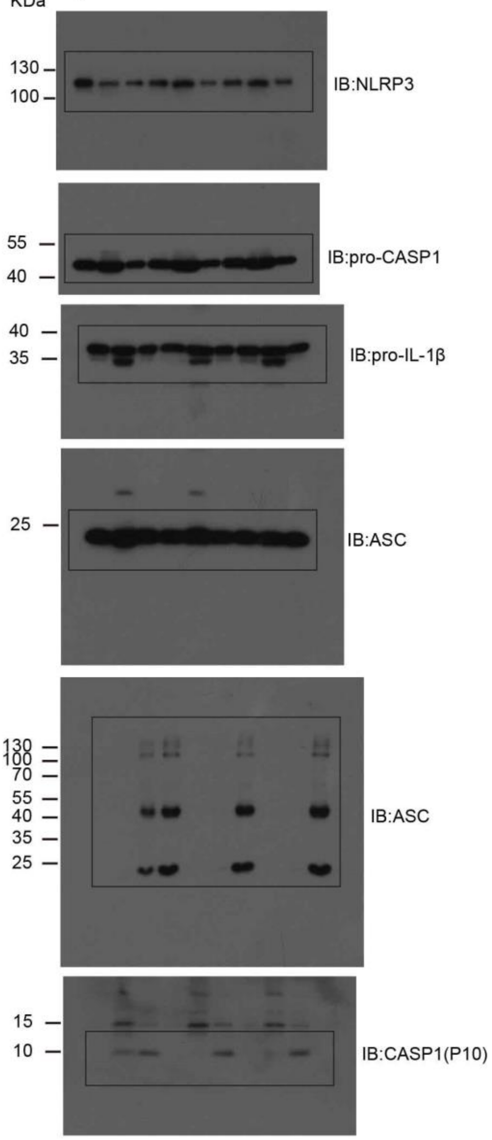

Figure 3f

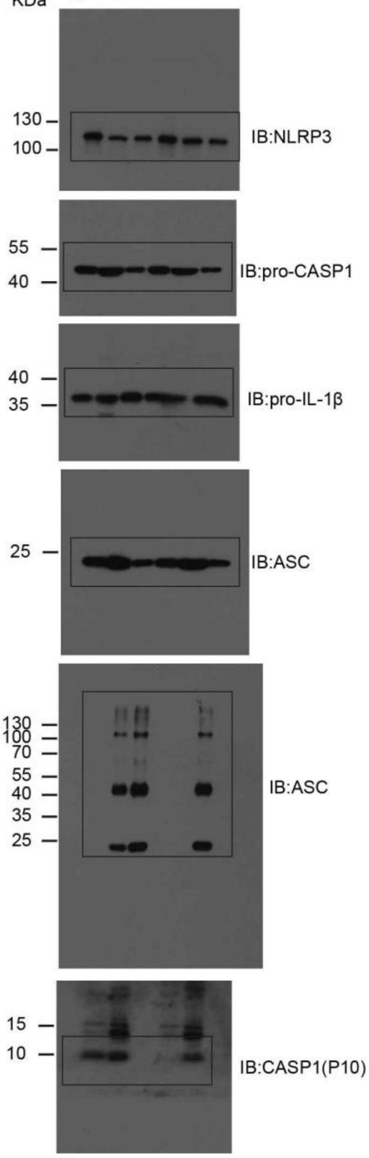

Supplementary Figure 9. (continued below)

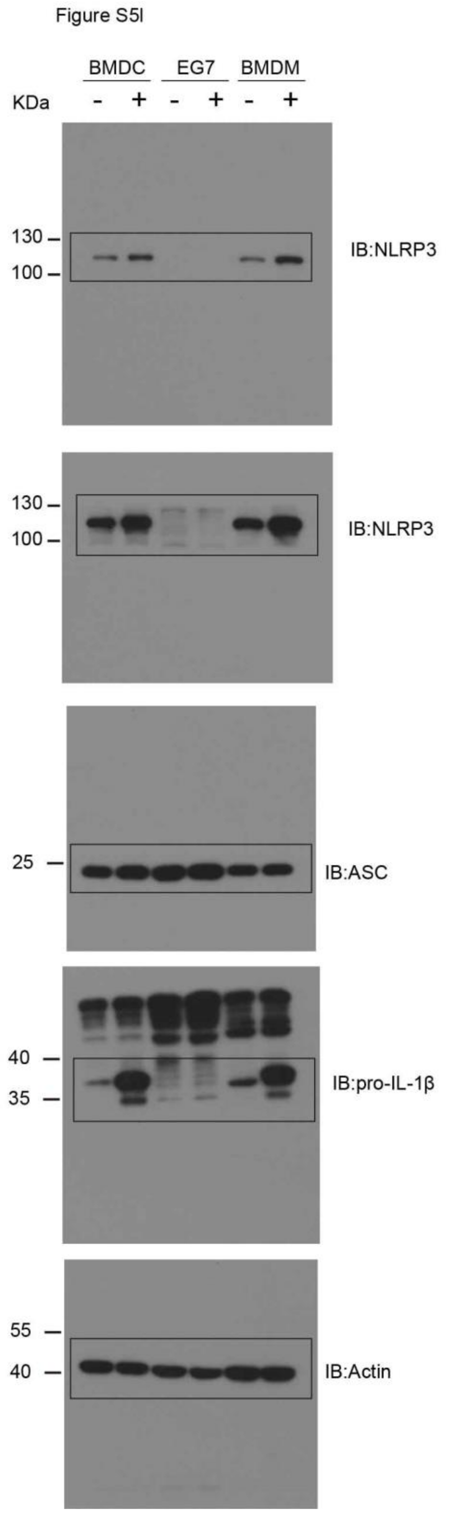

Supplementary Figure 9. Non-cropped versions of the western blots shown in Figure 1c, 1i, Figure 2d,2h, Figure 3b,3f and Supplementary Figure 5l.

Supplementary Table 1.

Gene	Direction	Primer sequence (5'->3')
mouse granzyme A	forward	TTTCATCCTGTAATTGGACTAA
	reverse	GCGATCTCCACACTTCTC
mouse granzyme B	forward	CCTCCTGCTACTGCTGAC
	reverse	GTCAGCACAAAGTCCTCTC
mouse granzyme C	forward	TTCTCCTGACCCTACTTCTG
	reverse	TGTTAGCACGAATTTGTCTC
mouse granzyme M	forward	AGCAGGCAACAGATTTGAGAC
	reverse	CATGTATGGGCGGGAGTG
mouse granzyme K	forward	CATCCAGTACCGCAGCAAG
	reverse	GAGAGTGGCCTCTGGGAAA
mouse perforin	forward	GAGAAGACCTATCAGGACCA
	reverse	AGCCTGTGGTAAGCATG
mouse Itpr1 (IP3R1)	forward	CTCTGTATGCGGAGGGATCTAC
	reverse	GCGGAGTATCGATTCATAGGA
mouse Itpr2 (IP3R2)	forward	CTTCCTCTACATTGGGGACATC
	reverse	GGCAGAGTATCGATTCATAGGG
mouse Itpr3 (IP3R3)	forward	AGCCAAGCAGACTAAACAGGAC
	reverse	GCCGCTTGTTCACAGTTAAGTA
mouse RyR1	forward	GCCTTTGACGTGGGATTACAG
	reverse	CCCCAACTCGAACCTTCTCTC
mouse RyR2	forward	ACGGCGACCATCCACAAAG
	reverse	AAAGTCTGTTGCCAAATCCTTCT
mouse RyR3	forward	ACCAGCAGGAGCAAGTACG
	reverse	GGGGTCGTGTCAAAGTAGTCA
mouse Il6	forward	GATGGATGCTACCAAACTGGAT
	reverse	CCAGGTAGCTATGGTACTCCAGA
mouse Il2	forward	CCTGAGCAGGATGGAGAATTACA
	reverse	TCCAGAACATGCCGCAGAG
mouse TNF	forward	TCTTCTCATTCCTGCTTGTGG
	reverse	GGTCTGGGCCATAGAACTGA
mouse IFN-γ	forward	CAGCAACAGCAAGGCGAAA
	reverse	CTGGACCTGTGGGTTGTTGAC
mouse Rpl13a	forward	GGGCAGGTTCTGGTATTGGAT
	reverse	GGCTCGGAAGTGGTAGGGG

Supplementary Table 1. PCR Primers for RNA quantification related to experimental procedures
